# Cerebral blood flow pulsatility and cerebral artery stiffness acutely decrease during hemodialysis

**DOI:** 10.14814/phy2.15595

**Published:** 2023-02-17

**Authors:** Mathilde Paré, Hasan Obeid, Lawrence Labrecque, Audrey Drapeau, Patrice Brassard, Mohsen Agharazii

**Affiliations:** ^1^ CHU de Québec Research Center, L'Hôtel‐Dieu de Québec Hospital Québec City Quebec Canada; ^2^ Research Center of the Institute Universitaire de Cardiologie et de Pneumologie de Québec Québec City Quebec Canada; ^3^ Department of Kinesiology, Faculty of Medicine Université Laval Québec City Quebec Canada; ^4^ Division of Nephrology, Faculty of Medicine Université Laval, Québec Québec City Quebec Canada

**Keywords:** arterial stiffness, cerebral pulsatility index, end‐stage kidney disease, hemodialysis, middle cerebral artery mean blood velocity, pulse wave velocity

## Abstract

End‐stage kidney disease (ESKD) is associated with increased arterial stiffness and cognitive impairment. Cognitive decline is accelerated in ESKD patients on hemodialysis and may result from repeatedly inappropriate cerebral blood flow (CBF). The aim of this study was to examine the acute effect of hemodialysis on pulsatile components of CBF and their relation to acute changes in arterial stiffness. In eight participants (age: 63 ± 18 years, men: 5), CBF was estimated using middle cerebral artery blood velocity (MCAv) assessed with transcranial Doppler ultrasound before, during, and after a single hemodialysis session. Brachial and central blood pressure, along with estimated aortic stiffness (eAoPWV) were measured using an oscillometric device. Arterial stiffness from heart to MCA was measured as the pulse arrival time (PAT) between electrocardiogram (ECG) and transcranial Doppler ultrasound waveforms (cerebral PAT). During hemodialysis, there was a significant reduction in mean MCAv (−3.2 cm/s, *p* < 0.001), and systolic MCAv (−13.0 cm/s, *p* < 0.001). While baseline eAoPWV (9.25 ± 0.80 m/s) did not significantly change during hemodialysis, cerebral PAT increased significantly (+0.027 , *p* < 0.001) and was associated with reduced pulsatile components of MCAv. This study shows that hemodialysis acutely reduces stiffness of arteries perfusing the brain along with pulsatile components of blood velocity.

## Graphical abstract

This study assesses changes in cerebral blood velocity and regional arterial stiffness during hemodialysis. It shows an acute reduction of mean and pulsatile components of cerebral blood velocity, along with a reduction in the stiffness of arteries supplying the middle cerebral artery. This is the first study to assess cerebral artery stiffness in the setting of hemodialysis.

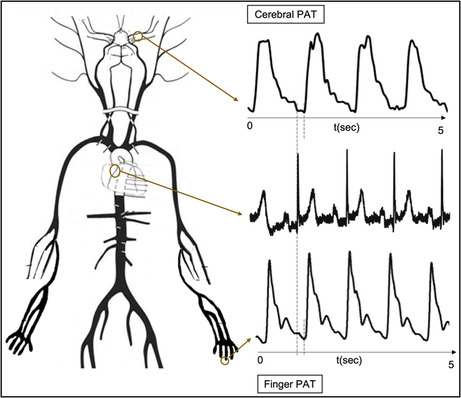

## 1 INTRODUCTION

Chronic kidney disease (CKD) affects an increasing number of individuals due to diabetes and aging of the population (Lv & Zhang, **2019**). In CKD, the risk of cardiovascular disease is increased above and beyond traditional cardiovascular risk factors (Go et al., **2004**; Parfrey & Foley, **1999**). Aortic stiffness is one of the nontraditional risk factors that may explain excess cardiovascular morbidity and mortality in CKD. Physiologically, stiffness of the arteries increases from the central arteries (i.e., aorta) to more peripheral arterial segments of the macrocirculation. This physiological gradient of arterial stiffness dampens flow pulsatility and protects the microcirculation against a highly pulsatile flow (Fortier & Agharazii, **2016**; London, **2018**). In CKD, aortic stiffening results in reduction of this physiological gradient and increased pulsatility of blood flow that primarily affects vascular beds of target organs, such as the brain and kidneys, that are characterized by high flow and low resistance (Avolio et al., **2018**; O'Rourke & Safar, **2005**). Over time, higher pulsatile blood flow is associated with cerebral microvascular dysfunction, likely as a result of unfavorable vascular remodeling processes (Rensma et al., **2020**). In turn, microvascular dysfunction has been linked to cognitive dysfunction (Shen et al., **2017**; Van Sloten et al., **2015**).

Cognitive impairment is prevalent in CKD, especially in patients with end‐stage kidney disease (ESKD) who require renal replacement therapy (Zammit et al., **2016**). This is in part not only due to advanced disease but also to factors relating to treatment. Indeed, ESKD patients treated by hemodialysis may undergo a faster decline in cognitive function than those treated by peritoneal dialysis (Ali et al., **2021**; Neumann et al., **2018**; Tian et al., **2019**). Among other explanations, repetitive exposure to hemodynamic stress and acute changes in the uremic toxin levels during hemodialysis could predispose patients to a more accelerated decline in cognitive function (Madero & Sarnak, **2011**; Wolfgram et al., **2015**).

During hemodialysis, large fluid shifts and secondary hemodynamic instability, along with changes in serum osmolality and pH, have been associated with intermittent reduction in brain perfusion and oxygenation (Sprick et al., **2020**; Wolfgram, **2019**). While cerebral blood flow and mean cerebral blood velocity have been measured during hemodialysis, very few studies have evaluated changes in pulsatile components of flow. Recently, Ghoshal et al. (**2021**) reported an increase in middle cerebral artery blood velocity (MCAv) pulsatility index during hemodialysis, which could contribute to cerebral damage in the long term. However, the study did not provide mechanistic insight into the determinants of increased pulsatility.

Acute changes in regional arterial stiffness could, in part, be responsible for intradialytic changes in pulsatility of cerebral blood velocity. While many investigators have studied changes in aortic stiffness before and after hemodialysis, there remains a lack of consensus regarding the direction of change, if any. In addition, few other arterial segments have been evaluated during hemodialysis, again, with conflicting result (Georgianos et al., **2011**; Mourad et al., **2004**; Şahin Yildiz et al., **2015**). To the best of our knowledge, acute changes in stiffness of extracranial or intracranial arteries perfusing the brain have never been investigated. During hemodialysis, stiffness of these arteries could be altered as a result of acute reduction in intravascular volume, or alterations in sympathetic tone, among other mechanisms (Boero et al., **2001**).

Therefore, during a single hemodialysis session, we aimed to examine (1) the changes in both mean and pulsatile components of cerebral blood velocity, (2) the changes in arterial stiffness indices of various arterial segments, including arteries perfusing the brain, and (3) the relationship between mean and pulsatile indices of blood velocity and regional markers of arterial stiffness. We hypothesized that (1) mean MCAv decreases while pulsatile components of blood velocity increase during hemodialysis, (2) indices of aortic and cerebral stiffness decrease during hemodialysis while peripheral stiffness increases, and (3) during hemodialysis, pulsatile components of blood velocity are associated with regional markers of arterial stiffness.

## 2 METHODS

### 2.1 Ethics Statement

All participants provided written informed consent prior to participating in the investigation, and the study was approved by the Comité d'éthique de la recherche du Centre Hospitalier Universitaire de Québec (CHUQ) – Université Laval (CER: 21180) according to the principles established in the Declaration of Helsinki (except for registration in a database) and the Canadian Government Tri‐Council Policy Statement (TCPS2) for integrity in research.

### 2.2 Study population

**FIGURE 1:**
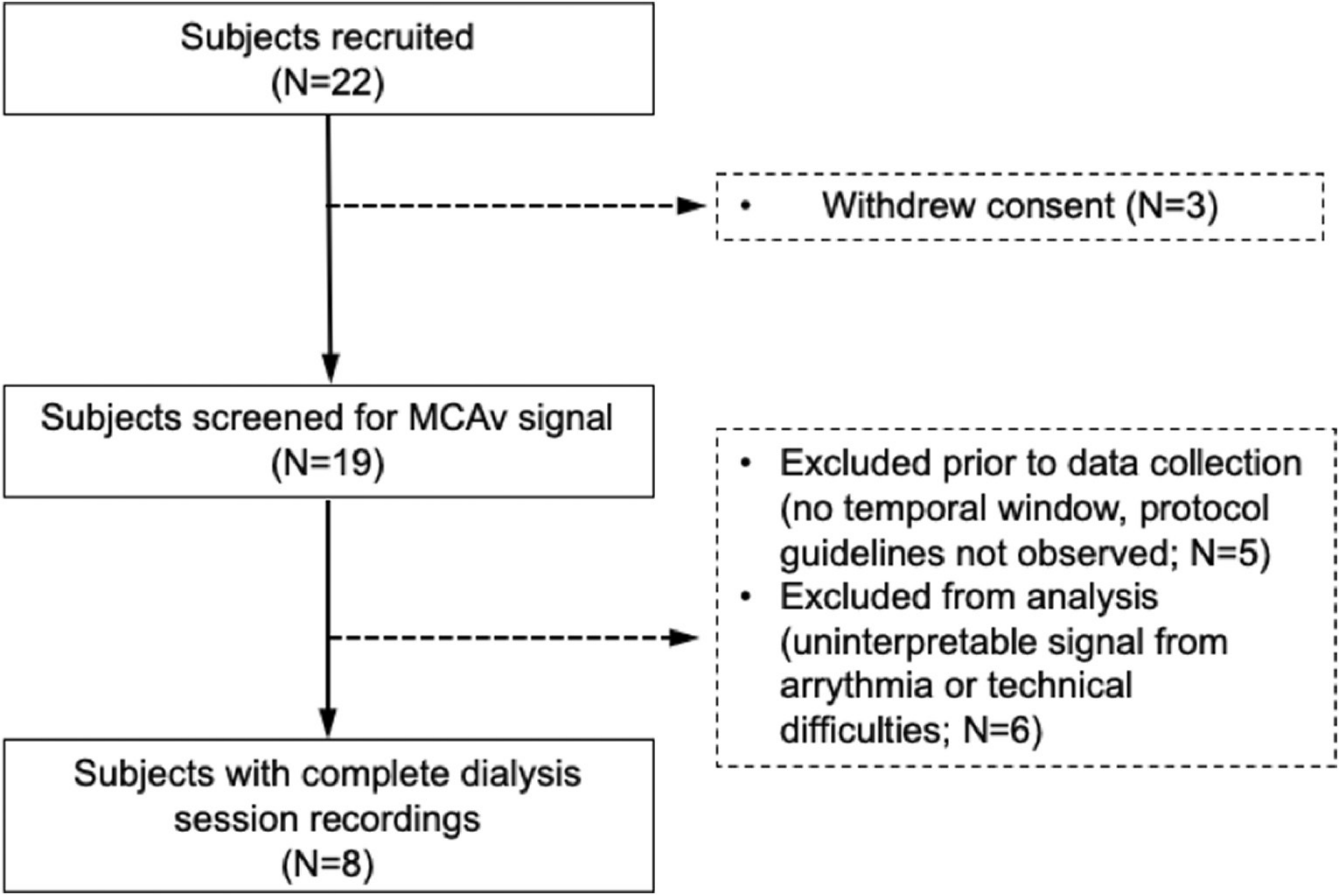
Study flowchart: This flowchart outlines the recruitment process for this pilot study.

Twenty‐two participants were recruited from the Dialysis Unit of the CHUQ –L'Hôtel‐Dieu de Québec between June and September 2018. Patients were eligible for the study if they met the following inclusion criteria: >18 years, ESKD defined as an estimated glomerular filtration rate (eGFR) <15 mL/min/1.73m^2^, and prevalent hemodialysis >3 months, with a single pooled Kt/V ≥ 1.3 indicating effective hemodialysis, as calculated from urea clearance. Patients with severe carotid artery stenosis (>70%, Ferguson et al., **1999**), atrial fibrillation, history of prevalent cognitive decline, neurologic diseases, or past cerebrovascular event, as established from medical records, or lacking an adequate temporal window for transcranial Doppler ultrasound (TCD) transmission, were excluded from the study. Participants who failed to follow pre‐protocol guidelines were also excluded (study flow chart presented in Figure 1). These inclusion and exclusion criteria were used to ensure the reliability of cerebral and central hemodynamic measurements. After exclusions, eight participants had complete data and composed the final sample.

Baseline demographic and clinical patient characteristics were collected from patient medical charts and are presented in Table 1. The etiology of ESKD in participants were diabetic nephropathy (*N* = 2), hypertensive nephroangiosclerosis (*N* = 2), glomerulonephritis (*N* = 1), polycystic kidney disease (*N* = 1), tuberous sclerosis (*N* = 1), and unknown origin (*N* = 1). Arterial hypertension was defined as either documented prevalent arterial hypertension or patients taking antihypertensive medication. Cardiovascular disease was defined as either a past cardiovascular event or documented coronary obstruction.

**TABLE 1:** Participant baseline characteristics.

Parameters	Value
Demographic characteristics	
Age (years)	63 ± 18
Male (*N*, %)	5 (63)
Arterial hypertension (*N*, %)	4 (50)
Diabetes (*N*, %)	4 (50)
CVD (*N*, %)	4 (50)
Smoking (*N*, %)	1 (13)
BMI (kg/m^2^)	28.9 ± 5.0
Body weight (kg)	79.2 ± 16.5
Biochemical	
Hb (g/L)	107.9 ± 19.3
Albumin (g/L)	33.5 ± 3.2
Calcium (mmol/L)	2.3 ± 0.2
Phosphate (mmol/L)	1.5 ± 0.5
PTH (mmol/L)	193 [153–573]
Medication	
ASA	5 (63)
Statin	4 (50)
ACEi/ARB	3 (38)
Beta‐blockers	5 (63)
Calcium channel blockers	2 (25)
Diuretics	1 (13)
Nitrates	2 (25)
Dialysis specific characteristics	
Dialysis vintage (months)	27.5 [7.3–45.8]
sp KT/V	1.7 ± 0.2
Ultrafiltration volume (kg)	2.1 [0.9–2.4]
Hemodialysis vascular access	
Arteriovenous fistula	4 (50)
Central venous catheter	4 (50)

*Note*: Values are mean ± SD, *N* (%), or median (25th–75th percentile).

Abbreviations: ACEi, angiotensin‐converting enzyme inhibitor; ARB, angiotensin receptor blockers; ASA, acetylsalicylic acid; BMI, body mass index; CRP, C‐reactive protein; CVD, cardiovascular disease; Hb, hemoglobin; PTH, parathyroid hormone; sp KT/V, single pool KT/V; TG, triglyceride.

### 2.3 Experimental protocol

**FIGURE 2:**
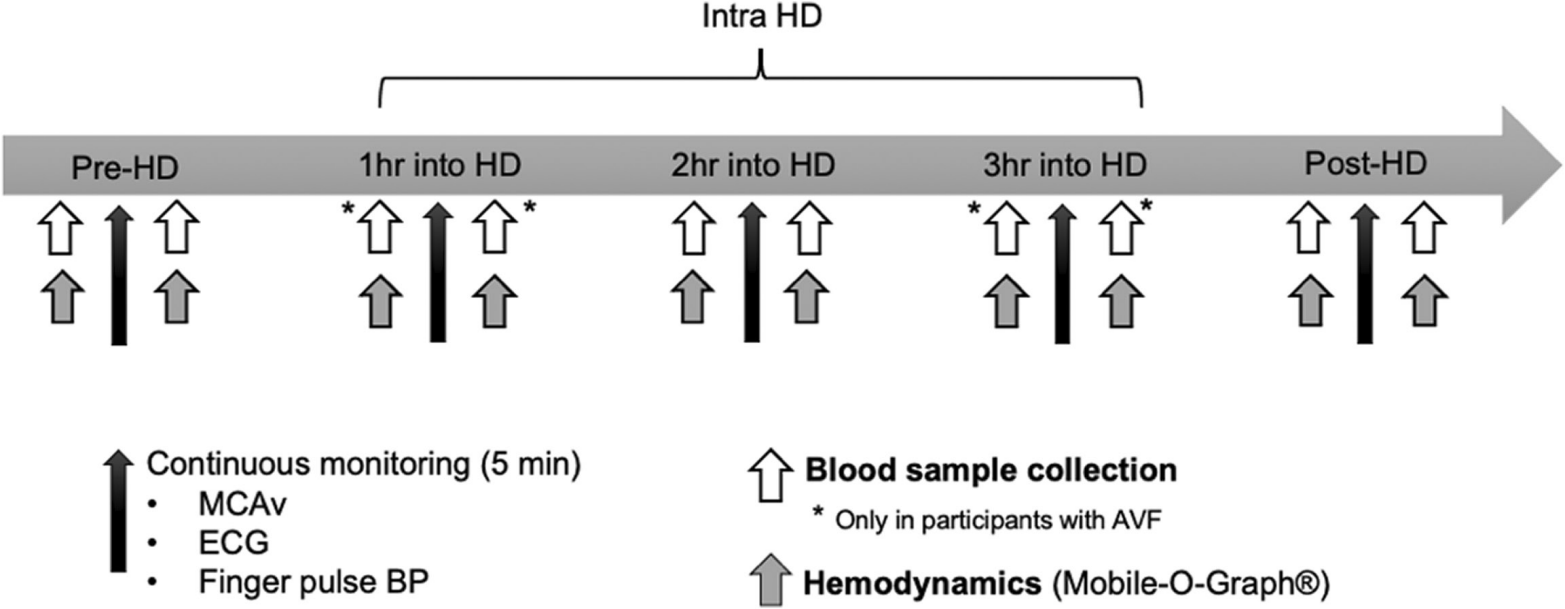
Study protocol: Time into hemodialysis is shown as a horizontal arrow. Intra‐HD indicates the averaged data obtained at each hour during hemodialysis. Vertical arrows represent timing of measurements. Thin dark vertical arrows indicate timing of continuous monitoring over 5 min periods. AVF, arteriovenous fistula; BP, Blood pressure; ECG, electrocardiogram; MCAv, middle cerebral artery blood velocity.

The experimental protocol for this study is graphically presented in Figure 2. Prior to participation, participants were instructed to avoid caffeine or alcohol consumption within 24 h of the study, to refrain from exercise training or smoking 12 h prior to the study, and to arrive 1 h before their scheduled hemodialysis onset time. They were weighed, measured, and body mass index was calculated as body mass (kg)/height^2^ (m). Participants were then instrumented with an electrocardiogram (ECG), a continuous blood pressure (BP) monitoring finger cuff, a brachial ambulatory BP monitoring system, and the TCD prior to hemodialysis onset. The participants remained connected to the probes and sat in a semi‐recumbent position throughout hemodialysis.

For the study, each participant underwent their regular 4 h hemodialysis session, as prescribed by their nephrologist. Hemodialysis was performed using FX‐100 membranes (*N* = 4), LO‐20 (*N* = 2), and Evodial 1.6 (*N* = 2). Vascular access was achieved through an arteriovenous fistula in half (*N* = 4) of the participants, while the other half had central venous (right jugular vein) catheters. A bicarbonate‐based buffer dialysis solution was used, with sodium concentrations [Na^+^] of 135–146 mmol/L, potassium concentrations of 2–3 mmol/L, a bicarbonate concentration of 31 or 34 mmol/L, a calcium concentration of 1.25–1.5 mmol/L, dextrose 5.5 mmol/L, blood flow of 350–400 mL/min, and dialysate flow rate of 500–800 mL/min.

During hemodialysis, periodic cerebrovascular assessments consisting of 5 min of continuous monitoring of MCAv, mean arterial pressure (MAP), and ECG activity were performed. These periodic assessments were completed within 30 min prior to hemodialysis start (pre‐hemodialysis), after 1 h, 2 h, and 3 h of hemodialysis, and finally within 15 min of hemodialysis completion (post‐hemodialysis). Immediately before and after each 5 min monitoring period, central hemodynamic parameters of interest (central BPs and estimated aortic stiffness), as well as blood gases were measured. Given that these values did not significantly differ, they were averaged for each hour of hemodialysis. Finally, participants remained awake with their eyes opened and refrained from talking, or coughing, as to minimize the impact of increased intra‐abdominal pressure on physiological measurements.

## 3 MEASUREMENTS

### 3.1 Cerebral blood flow

Cerebral blood flow was estimated by monitoring MCAv using standardized procedures to identify the MCA (Willie et al., 2011) with a 2 MHz pulsed TCD probe (Doppler Box; Compumedics DWL, Inc.). The probe was fixed in place with a commercial headframe (DWL USA) and adhesive ultrasonic gel (Tensive, Parker Laboratory). Systolic MCAv (syst MCAv) and diastolic MCAv (diast MCAv) were defined as maximum and minimum of the blood velocity waveform, respectively. Mean MCAv was calculated as the area under the curve of the blood velocity waveform divided by the duration of the cardiac cycle. Pulsed MCAv (P‐MCAv; syst MCAv–diast MCAv) and pulsatility index (cerebral PI; syst MCAv–diast MCAv/mean MCAv) were calculated. The cerebral resistive index (cerebral RI) was calculated as syst MCAv–diast MCAv/syst MCAv. Cerebrovascular conductance index (CVCi), a reflection of cerebrovascular tone (Lautt, **1989**) was calculated as the ratio of mean MCAv/MAP. Conversely, cerebrovascular resistance index (CVRi) was calculated as the ratio of MAP/mean MCAv.

A five‐lead ECG remained in place during the entire study. Beat‐to‐beat BP was assessed with the volume clamp method using a finger cuff, which was installed on a finger of the hand contralateral to the arteriovenous fistula, when present, and referenced to the level of the heart using a height correcting unit for BP correction (Nexfin, Edwards Lifesciences). While systolic and diastolic finger BP were defined as peak and minimum of the pressure waveform, respectively, MAP was obtained by integration of the pressure waveform divided by the duration of the cardiac cycle.

Continuous MCAv, BP, and ECG signals were analog‐to‐digital converted at 1000 Hz via an analog‐to‐digital converter (Powerlab 16/30 ML880; ADInstruments) and stored for subsequent analysis using commercially available software (LabChart version 7.1; ADInstruments). A representative 1 min sequence was selected from the continuous raw ECG, finger photoplethysmography (PPG), and MCAv data at each time point of interest, that is, pre‐hemodialysis, every hour during hemodialysis, and post‐hemodialysis.

Using MATLAB (R2018b, MathWorks), the net signals were filtered to obtain a clear signal. The recorded signal sequences were passed through a low‐pass filter characterized by a straight‐line phase. The same procedure of filtering and smoothing was applied to both MCAv and PPG signals, given that differences in signal treatment might have otherwise induced phase shifts. Setting the cut‐off frequencies of the filter requires prior knowledge of the signal and noise frequency spectra so that the filter's frequency response can be determined. This information was obtained through fast‐Fourier transform, where the raw signals were transformed from the time domain to the frequency domain, in order to determine the frequency of the noisy part to be eliminated by the filter. A 45 Hz threshold was selected.

### 3.2 Arterial stiffness indices

**FIGURE 3:**
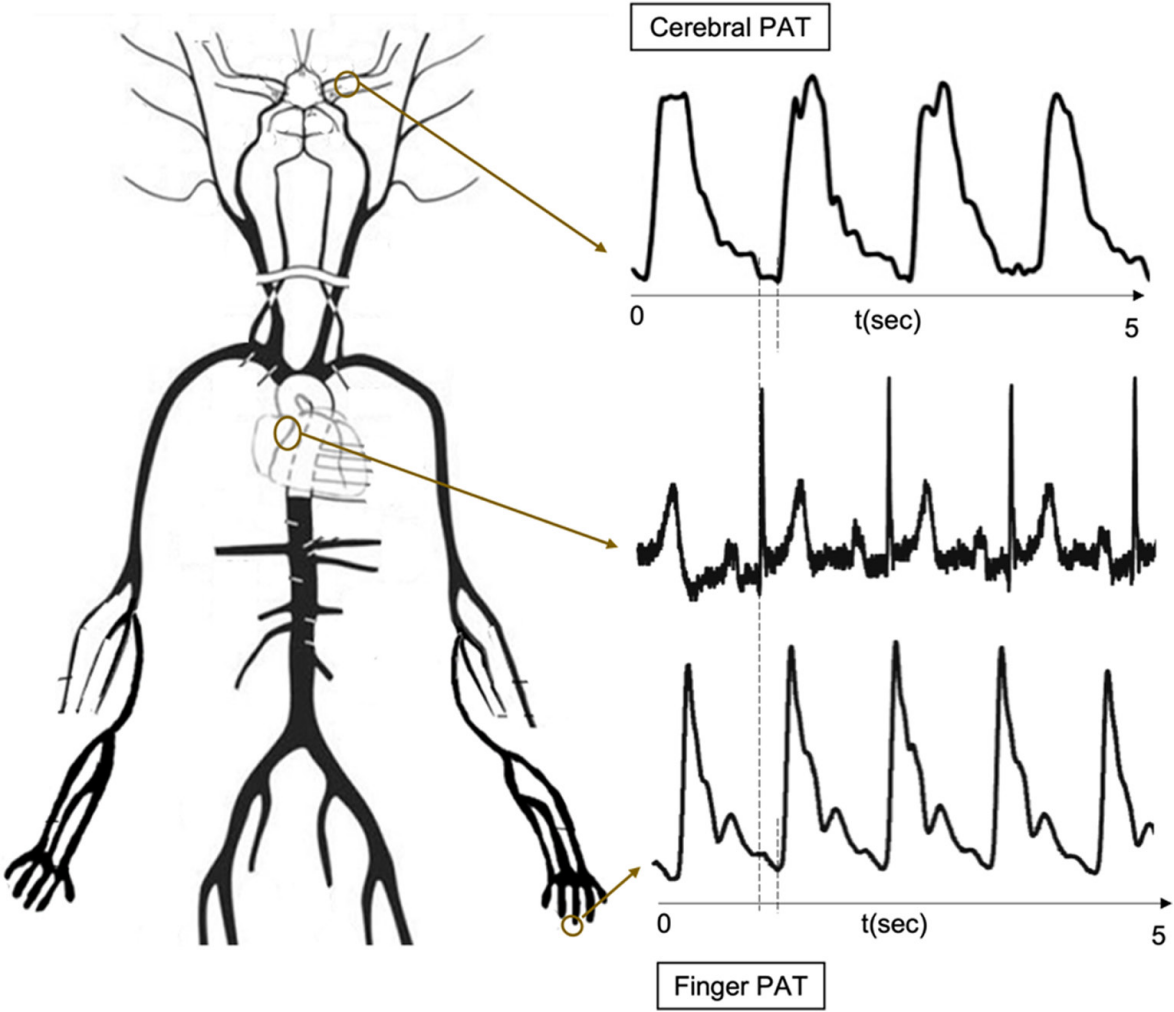
Visual representation of cerebral and finger pulse arrival time assessment. The left panel of the figure provides a visual representation of the arterial tree adapted from Obeid et al. (2017) Physiol Meas. Circles indicate location of measurements, that is, the middle cerebral artery, digital arteries, and heart. On the right panel, sample data taken at each location are represented along an *x‐*axis (time in seconds). Dotted vertical lines indicate the time differences between the peak of the ECG R wave and the foot of the middle cerebral artery waveform (upper third of right panel) and finger pulse waveform (lower third of right panel).

Indices of arterial stiffness included brachial pulse pressure (bPP), where bPP was the difference between brachial systolic (bSBP) and brachial diastolic BP (bDBP) and central pulse pressure (cPP), where cPP was the difference between central systolic blood pressure (cSBP) and central diastolic blood pressure (cDBP). Estimated aortic pulse wave velocity (eAoPWV) using Mobile‐O‐Graph (I.E.M. GmbH) provides an estimate of aortic stiffness, as previously described and validated (Hametner et al., **2013**). In addition, we used the pulse arrival time measured from the R wave of ECG to the foot of the MCA blood velocity waveform (cerebral PAT) and the PAT from R wave to the foot of the finger photoplethysmography pressure waveform (finger PAT). An increase in PAT is interpreted as a decrease in pulse wave velocity (i.e., arterial stiffness) along the arterial path from the heart to the MCA and to the digital arteries, respectively. The foot of both MCAv and finger pulse waveforms were identified as the timing of the maximum of the second derivatives of the waveforms, after smoothing the curves by a triangle moving average filter. The method used to assess cerebral and finger PAT is visually summarized in Figure 3.

### 3.3 Blood gas analysis

In all participants, capillary blood samples were drawn before and after hemodialysis. During hemodialysis, arterial blood was accessible through the patient's arteriovenous fistula (*N* = 4) and was drawn each hour during hemodialysis. In those with a central venous catheter, intra‐hemodialysis capillary blood was drawn at the 2 h within hemodialysis time point, to limit uncomfortable fingertip blood draws.

Blood samples were analyzed using a blood gas analysis system (ABL835 Flex, Radiometer Medical ApS). Briefly, this blood gas analysis system provides reliable readings of pH and PCO_2_ (Torr), where [HCO_3_^−^] is calculated from measured PaCO_2_ and pH, using the Henderson–Hasselbalch equation (Hoiland et al., **2019**). The pKa (i.e., − log of the acid dissociation constant) at 37.0°C of 6.1 and the solubility factor for dissolved CO_2_ plus carbonic acid (H_2_CO_3_) at 37.0°C in plasma of 0.0314 mEq × L^−1^ per Torr PaCO_2_ were used: pH = 6.1 + log [HCO_3_^−^]/(0.0314 × PaCO_2_).

### 3.4 Statistical analysis

Data are reported as mean ± standard deviation, *n* (%) or median (25th–75th percentiles), and mean (95% CI). To account for intraindividual changes and repeated measures analysis, we used generalized estimating equations (GEE) using three time levels: before hemodialysis, during hemodialysis, and after hemodialysis. A robust estimator of covariance matrix was used. Values of the estimates are reported ± standard errors whenever a GEE was used. The *p*‐values were corrected using sequential Bonferroni for multiple comparisons. Using this approach, we examined the changes in hemodynamic variables in response to hemodialysis. We then examined the relationship between MCAv parameters and clinical, hemoglobin, PCO_2_, and arterial stiffness indices. Data analysis were performed using SPSS software (version 26.0; SPSS Inc.). Statistical significance was set a priori to an alpha value <0.05.

## 4 RESULTS

### 4.1 Baseline characteristics of study participants

Demographic and clinical characteristics of participants are summarized in Table 1. Briefly, participants were typical hemodialysis patients with an average age of 63 ± 18 years, BMI of 29 ± 5 kg/m^2^, and had been on hemodialysis for a median period of 28 (7–46) months, with the expected high frequency of diabetes, arterial hypertension, cardiovascular disease, and cardiovascular medication. All patients suffered from anemia with a mean hemoglobin concentration of 108 ± 19 (g/L), ranging from 78 to 116 g/L. Other biological parameters are presented in Table 1. The total ultrafiltration volume during hemodialysis was 2.1 L (0.9–2.4).

Baseline cerebral hemodynamics along with central and peripheral BP are reported in Table 2. Baseline aortic stiffness, as estimated by eAoPWV was 9.25 ± 0.80 m/s, while cerebral PAT and finger PAT were 0.17 ± 0.01 s and 0.19 ± 0.01 s, respectively.

**TABLE 2:** Intradialytic changes in cerebral hemodynamics and regional arterial stiffness.

	Pre‐HD	Intra‐HD	Pre‐intra HD, *p*‐value	Post‐HD	Pre‐post HD, *p*‐value
Brachial blood pressure (mm Hg)					
bSBP	130.8 ± 6.6	121.1 ± 5.6	0.051	134.6 ± 6.1	0.343
bDBP	78.5 ± 3.5	75.1 ± 2.7	0.170	81.6 ± 3.8	0.422
bPP	53.6 ± 5.2	46.5 ± 4.2	0.140	51.4 ± 4.4	0.655
Central blood pressure (mm Hg)					
cSBP	117.5 ± 5.5	110.0 ± 5.1	0.107	119.9 ± 5.9	0.556
cDBP	77.6 ± 3.1	76.2 ± 2.8	0.470	84.0 ± 3.2	0.063
cPP	40.7 ± 4.1	34.1 ± 3.6	0.116	34.4 ± 3.5	0.093
Cerebral hemodynamics					
Syst MCAv (cm/s)	101.9 ± 8.9	88.9 ± 7.4	0.016	89.5 ± 7.2	0.038
Diast MCAv (cm/s)	42.3 ± 3.8	38.7 ± 1.7	0.226	38.0 ± 2.1	0.226
Mean MCAv (cm/s)	73.9 ± 4.6	70.7 ± 4.9	<0.001	67.8 ± 5.0	<0.001
P‐MCAv (cm/s)	59.6 ± 7.8	50.2 ± 7.7	0.011	51.9 ± 7.1	0.051
Cerebral PI	0.80 ± 0.05	0.72 ± 0.10	0.722	0.79 ± 0.11	0.879
Cerebral RI	0.58 ± 0.04	0.54 ± 0.04	<0.001	0.56 ± 0.04	0.586
MAP (mm Hg)	93.7 ± 5.8	85.3 ± 4.3	0.009	78.8 ± 4.4	<0.001
CVCi (cm/s/mm Hg)	0.80 ± 0.05	0.83 ± 0.06	0.192	0.86 ± 0.07	0.115
CVRi (mm Hg/cm/s)	1.28 ± 0.06	1.24 ± 0.08	0.292	1.22 ± 0.08	0.276
Other parameters					
HR (bpm)	61.7 ± 7.2	68.3 ± 3.8	0.092	71.1 ± 4.5	0.025
PCO_2_ (mm Hg)	41.8 ± 1.0	39.4 ± 1.1	0.032	42.5 ± 1.1	0.463
[HCO_3_^−^] (mEq L^−1^)	20.8 ± 0.4	26.3 ± 0.5	<0.001	27.1 ± 0.5	<0.001

*Note*: Data are presented as estimated marginal mean ± standard error; ***P***‐values for simple comparisons to baseline, generalized estimating equation models.

Abbreviations: [HCO_3_−], bicarbonate anion concentration; bDBP, brachial diastolic blood pressure; bMAP, brachial mean blood pressure; bPP, brachial pulse pressure; bSBP, brachial systolic blood pressure; cDBP, central diastolic blood pressure; Cerebral PAT, electrocardiogram to cerebral pulse arrival time; Cerebral PI is syst MCAv–diast MCAv/(Mean MCAv); Cerebral RI is syst MCAv–diast (MCAv)/syst MCAv; cPP, central pulse pressure; cSBP, central systolic blood pressure; CVCi, cerebrovascular conductance index; CVRi, cerebrovascular resistance index; Diast MCAv, Diastolic MCAv; eAoPWV, estimated aortic pulse wave velocity; Finger PAT, electrocardiogram to finger pulse arrival time; HR, heart rate; MAP, mean arterial pressure (digital photoplethysmography); MCAv, middle cerebral artery blood velocity; PCO_2_, partial pressure of CO_2_; P‐MCAv, pulsed MCAv; Syst MCAv, systolic MCAv.

### 4.2 Hemodynamic changes during hemodialysis

Table 2 shows the changes in central and cerebral hemodynamic parameters during hemodialysis. While there was a decrease in bSBP (−9.7 mm Hg, 95% CI: −19.3 to 0.03, *p* = 0.051) of borderline statistical significance during hemodialysis, cSBP (−7.5 mm Hg, 95% CI: −17.0 to 1.9, *p* = 0.107) did not significantly decrease. Changes in bDBP, cDBP, bPP, and cPP were not statistically significant.

**FIGURE 4:**
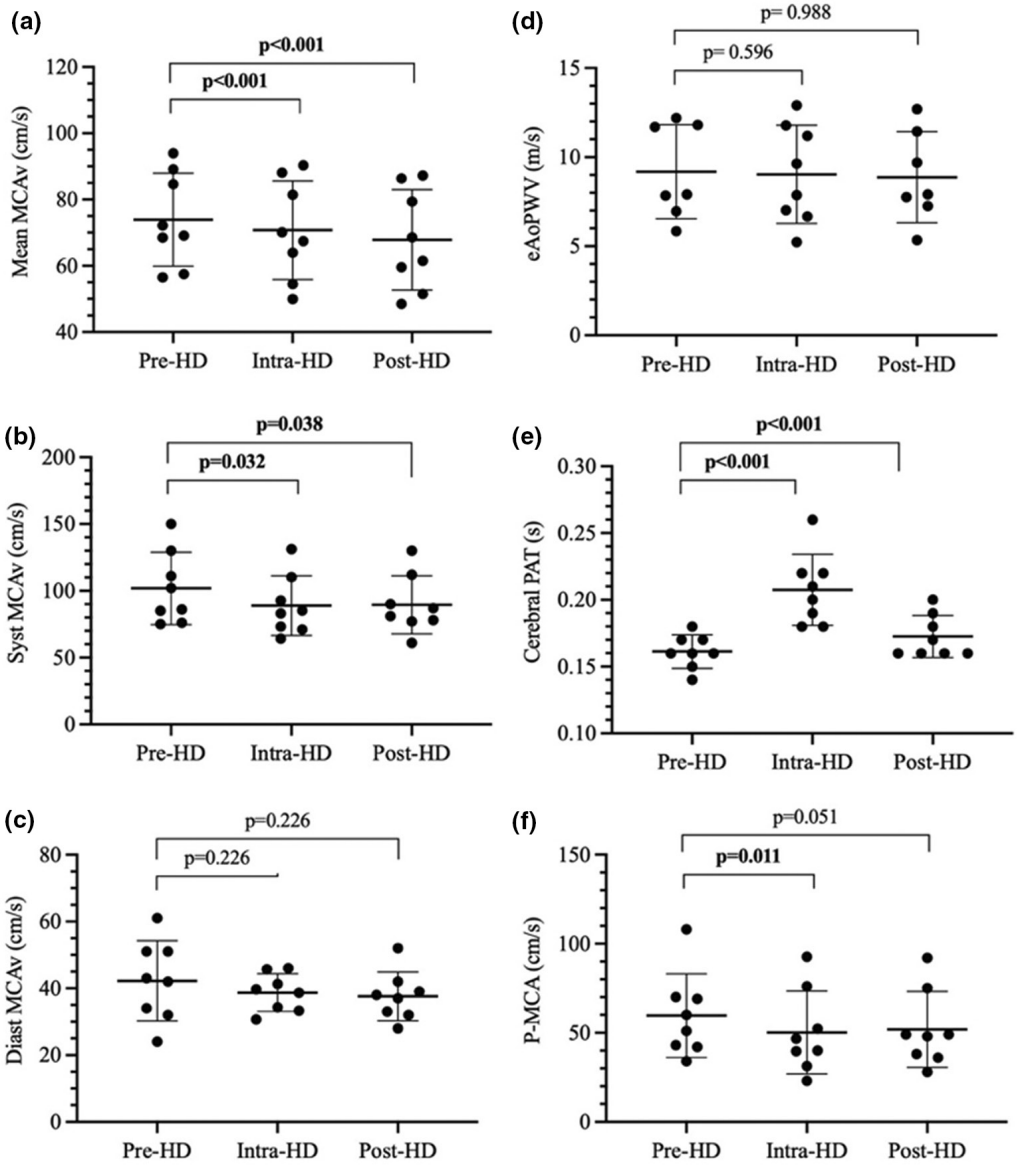
Cerebral blood velocities, pulsatility, and stiffness during hemodialysis. Each panel presents the evolution of a cerebral or systemic hemodynamic variable during hemodialysis. Time into hemodialysis is presented on the *x*‐axis. Data obtained Pre‐HD were collected just before dialysis initiation. Data points presented as Intra‐HD are averaged from measurements taken at 1, 2, and 3 h into hemodialysis. Data marked as Post‐HD was collected within 15 min after hemodialysis. Diast MCAv, diastolic middle cerebral artery velocity; eAoPWV, estimated aortic pulse wave velocity; cerebral PAT, pulse arrival time between the peak of the ECG R wave and the foot of the transcranial Doppler ultrasound waveform; Mean MCAv, mean middle cerebral artery velocity; P‐MCAv, pulsed middle cerebral artery velocity; Syst MCAv, systolic middle cerebral artery velocity. *p*‐values indicate the level of statistical significance of the change relative to the Pre‐HD baseline and is obtained from generalized estimating equation models.

As presented in Table 2 and illustrated in Figure 4, mean MCAv decreased during hemodialysis (−3.2 cm/s, 95% CI: −4.4 to −2.0, *p* < 0.001), and remained lower after hemodialysis (−6.1 cm/s, 95% CI: −7.4 to −4.7, *p* < 0.001). Syst MCAv was significantly reduced during hemodialysis (−13.0 cm/s, 95% CI: −23.4 to −3.2, *p* = 0.016) and remained so following the end of hemodialysis (−12.4 cm/s, 95% CI: −24.2 to −0.6, *p* = 0.038). Diast MCAv remained relatively unchanged during hemodialysis (−3.6 cm/s, 95% CI: −8.1 to 0.9, *p* = 0.226) and after hemodialysis (−4.4 cm/s, 95% CI: −10.7 to 1.9, *p* = 0.226), which resulted in a reduction of P‐MCAv during (−9.4 cm/s, 95% CI: −16.1 to −2.7, *p* = 0.011) and after hemodialysis (−7.8 cm/s, 95% CI: −15.6 to −1.6, *p* = 0.051). When adjusting for bSBP and cSBP, the reduction in syst MCAv was no longer statistically significant during hemodialysis (−9.7 cm/s, 95% CI: −18.9 to −0.5, *p* = 0.068 and − 9.2 cm/s, 95% CI: −18.4 to 0.01, *p* = 0.104, respectively) or after hemodialysis (−11.9 cm/s, 95% CI: −22.9 to −0.9, *p* = 0.068 and − 11.7 cm/s, 95% CI: −25.0 to 1.6, *p* = 0.104, respectively). However, there was a stronger association between mean MCAv with cSBP (slope = 0.370, *p* < 0.001) than bSBP (slope = 0.272, *p* = 0.005).

While the cerebral RI decreased significantly during hemodialysis (−0.04 s, 95% CI: −0.06 to −0.02, *p* < 0.001), it returned to baseline levels by the end of hemodialysis (−0.01 s, 95% CI: −0.05 to 0.03, *p* = 0.586). On the other hand, the cerebral PI, CVCi, and CVRi remained unchanged during the intervention (Table 2).

Finally, Table 2 also presents changes in bicarbonate concentration and PCO_2_. There was a small decrease in PCO_2_ during hemodialysis (−2.4 Torr, 95% CI: −4.36 to 0.44, *p* = 0.032), but this was no longer significant by the end of hemodialysis (+ 0.7 Torr, 95% CI: −1.26 to 2.66, *p* = 0.463).

### 4.3 Arterial stiffness indices during hemodialysis

Table 2 and Figure 4 report changes in arterial stiffness indices during the hemodialysis treatment. There were no significant changes in aortic stiffness, as measured by eAoPWV, during hemodialysis (−0.22 m/s, 95% CI: −0.63 to 0.19, *p* = 0.596) or after hemodialysis (−0.002 m/s, 95% CI: −0.59 to 0.25, *p* = 0.988). However, the cerebral PAT was significantly increased during hemodialysis (+0.042 s, 95% CI: 0.02–0.06, *p* < 0.001), and remained elevated by the end of hemodialysis (+0.013 s, 95% CI: 0.01–0.02, *p* < 0.001), suggesting a reduction of vascular stiffness in the segment from the heart to the MCA. This intradialytic increase in cerebral PAT remained significant after controlling for potential covariates including MAP, total fluid removal, and heart rate.

During hemodialysis, there was also an increase in finger PAT relative to baseline (+0.027 s, 95% CI: 0.02–0.04, *p* < 0.001), but there was no difference after hemodialysis (−0.001 s, 95% CI: −0.01 to 0.01, *p* = 0.848). This observation also suggests a transient reduction of vascular stiffness along the arterial path from the heart to the digital arteries.

### 4.4 Determinants of MCAv and its response during hemodialysis

At baseline, we found that for each reduction of 10 g/L of hemoglobin, the mean MCAv increased by 4.5 cm/s (95% CI: 2.3–6.7, *p* < 0.001). However, hemoglobin levels were not associated with the mean MCAv response to hemodialysis treatment (*p* = 0.405 for hemoglobin‐time interaction). Patients with diabetes had a higher mean MCAv by 20.1 cm/s (95% CI: 6.6–33.6, *p* = 0.003), but diabetes did not affect mean MCAv response to hemodialysis treatment (*p* = 0.984 for diabetes–time interaction). Furthermore, the impact of diabetes on mean MCAv was attenuated and no longer significant after correction for hemoglobin level (*p* = 0.081). Age, cardiovascular disease, sex, fluid removal, and PCO_2_ levels were not associated with baseline mean MCAv and its changes during or after hemodialysis treatment.

The relationships between indices of vascular stiffness and syst MCAv were further explored, along with their relationship to mean MCAv (Table 3). Syst MCAv was higher by 11 cm/s for every 1 m/s increase in eAoPWV (*p* < 0.001), and was higher by 4 cm/s (*p* = 0.023) and 4 cm/s (*p* = 0.015) for every 10 mm Hg increase in cPP and bPP, respectively. Syst MCAv was higher by 1 cm/s (*p* = 0.002) and 0.3 cm/s (*p* = 0.006) for every 0.01 s decrease in cerebral PAT and finger PAT, respectively. However, there were no associations between mean MCAv and any of the vascular stiffness indices.

**TABLE 3:** Determinants of blood velocity pulsatile components during hemodialysis.

Stiffness parameters	Mean MCAv (cm/s)		Systolic MCAv (cm/s)	
	Beta	*p*‐value	Beta	*p*‐value
bSBP (mm Hg)	−0.018	0.380	0.272	0.005
bPP (mm Hg)	−0.027	0.400	0.390	0.015
cSBP (mm Hg)	−0.001	0.954	0.370	<0.001
cPP (mm Hg)	0.006	0.812	0.368	0.023
eAoPWV (m/s)	−0.281	0.694	11.10	<0.001
Cerebral PAT (s)	−0.454	0.972	−86.70	0.002
Finger PAT (s)	−8.700	0.230	−31.78	0.006

	MCA pulsatility index ([Max‐Min]/mean)		P‐MCAv (cm/s) (Max‐Min)	
	Beta	*p*‐value	Beta	*p*‐value
bSBP (mm Hg)	0.003	0.060	0.152	0.048
bPP (mm Hg)	0.004	0.116	0.226	0.116
cSBP (mm Hg)	0.004	0.056	0.271	0.002
cPP (mm Hg)	0.004	0.051	0.302	0.010
eAoPWV (m/s)	0.062	0.003	7.600	<0.001
Cerebral PAT (s)	−0.779	0.015	−60.20	<0.001
Finger PAT (s)	0.056	0.855	−6.700	0.705

*Note*: Individual variable correlation coefficients obtained from generalized estimating equation models.

Abbreviations: bPP, brachial pulse pressure; bSBP, brachial systolic blood pressure; Cerebral PAT: electrocardiogram to cerebral pulse arrival time; cPP, central pulse pressure; cSBP, central systolic blood pressure; eAoPWV: estimated aortic pulse wave velocity; Finger PAT, electrocardiogram to finger pulse arrival time.

We also examined the relationship between pulsatile components of MCAv and indices of vascular stiffness. The results are shown in Table 3. It is worth underlining that P‐MCAv was most significantly associated with estimated aortic stiffness and stiffness of the vessels along the path from aorta to the MCA. In general, there was a greater consistency between MCAv pulsatility indices and central BP (cSBP and cPP) as compared to brachial BP (bSBP and bPP).

## 5 DISCUSSION

This study shows that mean MCAv acutely decreases during hemodialysis, a finding that is largely due to decreased intradialytic cSBP. It also demonstrates that arterial stiffness assessed between the heart and the MCA, acutely decreases during hemodialysis, and is significantly associated with pulsatile components of cerebral blood velocity. Regional arterial stiffness between the heart and the MCA was calculated using a novel method, based on physiological signals readily available at the bedside, which opens the door for use in future large‐scale studies.

### 5.1 Middle cerebral artery blood velocity is reduced during hemodialysis

In healthy individuals, CBF is regulated by a variety of complementary mechanisms to prevent damage from hypo‐ or hyperperfusion (Willie et al., **2014**). These include cerebral autoregulation, a mechanism that aims to maintain a relatively stable CBF, despite variations in arterial blood pressure. This mechanism is effective at limiting variation in CBF when changes in arterial BP are limited to 5–10 mm Hg (Brassard et al., **2021**). CBF is also influenced by arterial PCO_2_, a mechanism called cerebrovascular reactivity to CO_2_ (CVRCO_2_). Arterial bicarbonate concentration and pH also affect CBF, however, to a lesser degree than PCO_2_ (Caldwell et al., **2021**; Hoiland et al., **2019**).

In this study, mean MCAv was 73.9 ± 4.6 cm/s prior to hemodialysis start. This baseline mean MCAv was significantly associated with hemoglobin concentration, such that a 10 g/L decrease in hemoglobin equated a 5 cm/s increase in mean MCAv. While no reference values of CBF or MCAv have been reported for CKD and ESKD patients, we believe this finding is in line with available data. Indeed, previous authors have found that in ESKD, CBF starts to increase and is inversely proportional to hemoglobin concentration (Jiang et al., **2016**). Our relatively high baseline mean MCAv can further be explained by the heterogeneity of our sample, which included younger female participants, known to have higher MCAv (Alwatban et al., **2021**).

During hemodialysis, we observed a reduction in mean MCAv that was maintained after treatment. This finding is consistent with observations from previous groups that showed reductions either in direct measurements of CBF (Chung et al., **2016**; Gottlieb et al., **1987**; Holzer et al., **1981**; Polinder‐Bos et al., **2018**), or in estimations of flow from cerebral blood velocity during hemodialysis. Specifically, studies using TCD have shown decreases in MCAv ranging between 10% and 23% after hemodialysis (Findlay et al., **2019**; Hata et al., **1994**; Postiglione et al., **1991**; Stefanidis et al., **2005**). These findings were most often mediated by decreases in arterial BP. The roles of hemoglobin concentration, hematocrit, PCO_2_, and other metabolic factors in mediating changes in CBF or cerebral blood velocity were inconsistent between studies (Sprick et al., **2020**).

The reduction in mean MCAv observed during and after hemodialysis resulted from a reduction in syst MCAv, as there were no significant changes in diast MCAv. This reduction in syst MCAv was, in turn, largely mediated by the intradialytic reduction in systolic BP (both brachial and central). Central systolic BP, however, was more strongly associated with syst MCAv than bSBP. Previous studies have suggested that cerebral autoregulation is impaired in CKD patients when evaluated after a stroke or during hemodialysis (Castro et al., **2018**; MacEwen et al., **2017**). While our study was not designed to evaluate CBF regulatory mechanisms, we did observe an association between SBP and MCAv, suggesting that cerebral autoregulation may be impaired in ESKD patients during hemodialysis.

While the degree of anemia in ESKD patients was associated with baseline MCAv, it did not predict the response of MCAv to hemodialysis. Additionally, changes in mean MCAv during hemodialysis did not appear to be mediated by age, cardiovascular disease, sex, total fluid removal, or changes in blood gases. Indeed, after controlling for PCO_2_ and bicarbonate concentration, the reduction in mean MCAv remained statistically significant. Furthermore, neither PCO_2_ nor bicarbonate concentration were significantly associated with either baseline mean MCAv or changes in MCAv during hemodialysis. This lack of association between PCO_2_ and MCAv might suggest a reduction in CVRCO_2_ during hemodialysis, a finding in line with recent pilot work (Slessarev et al., **2021**). Indeed, Slessarev et al. observed a reduction in CVRCO_2_ in hemodialysis patients, when compared to CKD and healthy control participants.

### 5.2 Pulsatile components of cerebral blood velocity are reduced during hemodialysis

In addition to mean MCAv, we aimed to better understand the impact of hemodialysis on cerebral blood velocity pulsatility. We extracted syst MCAv, P‐MCAv, along with cerebral PI and cerebral RI. Contrary to our hypothesis, we found that during hemodialysis, pulsatile components of blood velocity, including, syst MCAv, P‐MCAv, and cerebral PI were significantly reduced compared to pre‐hemodialysis. While the absolute value of cerebral PI decreased during hemodialysis, this change was not statistically significant. Few previous studies have measured cerebral PI during hemodialysis. In 2002, Metry et al. (**2002**) had similarly observed no difference in cerebral PI in participants with decreased MAP during hemodialysis, but observed increased cerebral PI in those with stable MAP. These differences in findings may in part result from a higher calcium concentration (1.5 mmol/L) in the dialysis bath, acutely altering vascular tone and arterial stiffness (Mac‐Way et al., **2011**). In a more recent study, Ghoshal et al. (**2021**) observed a slight increase in cerebral PI during hemodialysis that was not statistically significant. Small sample size, along with limited information regarding study populations and dialysis characteristics, however limit comparison between studies.

In this study, the reduction in pulsatile components of cerebral blood velocity can in part be explained by the reduction in stiffness of the arteries perfusing the MCA. Indeed, pulsatile components of MCAv, including syst MCAv, P‐MCAv, and pulsatility index were significantly associated with estimated aortic stiffness and cerebral PAT, a finding in line with recent observations (Fico et al., **2022**).

### 5.3 Regional arterial stiffness is altered during hemodialysis

Arterial stiffness can be assessed over a given segment, where the length of the segment is divided by the pulse transit time (TT) to obtain a segmental PWV. Increased PWV corresponds to increased stiffness (O'Rourke & Mancia, **1999**). Stiffness of the aorta, brachial artery, and more recently digital arteries (Obeid et al., **2021**), have been assessed using noninvasive measurements of PWV. Assessing stiffness of cerebral arteries, however, poses a unique problem, as the rigid cranium prevents the use of pressure‐based methods to obtain reliable pulse waves further than the common carotid artery.

In 1994, Giller and Aaslid (**1994**) used ultrasound to measure the TT between internal carotid artery blood flow and MCAv waveforms. Their estimate of arterial length was 10 cm, which resulted in estimated _ICA‐MCA_PWV of 12.8 m/s. Similarly, Fu et al. (**2016**) assessed stiffness using duplex ultrasound and TCD between the common carotid artery and MCA, however estimating length based on distance between ultrasound probes and common carotid artery depth. They reported a mean _CCA‐MCA_ PWV of 4.99 ± 0.8 m/s. More recently, Balestrini et al. (**2020**), reported common carotid artery‐to‐MCA pulse wave TT of 0.013 ± 0.003 s in ischemic heart disease patients, that was not statistically different from that of control participants.

In this study, we assessed pulse arrival time measured between the ECG R wave and the foot of the MCA waveform and obtained a baseline cerebral PAT of 0.17 ± 0.01 s. We also assessed pulse arrival time between ECG R wave and the foot of the PPG waveform to obtain arterial stiffness between the heart and digital arterial and found an average finger PAT of 0.19 ± 0.01 s at baseline. In comparing with the cerebral PAT to that obtained by Balestrini et al. between the common carotid artery and MCA, we observed about a 10‐fold difference. This difference can be explained by the inclusion of cardiac pre‐ejection period into the measured time difference between ECG R wave and MCA waveform, that is, PAT (Beutel et al., **2021**). Given that neither method is normalized for arterial segment length, absolute TT or PAT values are of limited interest in comparing between groups. However, they provide valuable information when used to compare individuals to themselves when probes are kept in place such as in this study.

During hemodialysis, we observed a significant increase in cerebral PAT, that remained statistically significant after the end of hemodialysis. This increase in PAT suggests a reduction in arterial stiffness of the segment separating the heart and MCA, that is, including part of the ascending aorta, common and internal carotid arteries, and the early portion of the MCA. Similarly, we observed a transient increase in finger PAT during hemodialysis, that suggests reduced stiffness of arteries supplying the upper limb down to digital arteries. These reductions in regional arterial stiffness may in part result from decreased arterial BP, as BP is known to mechanically increase arterial stiffness. In addition, the reduction in stiffness could result from leukocyte activation as they come into contact with exogenous material from the dialysis membrane, releasing vasoactive mediators associated with vasodilation (Bowry et al., **2021**). This hypothesis, however, was not directly tested in our study.

### 5.4 Methodological considerations

This study has several strengths and limitations. First, it is the first study, to the best of our knowledge, to comprehensively describe the acute effect of hemodialysis on pulsatile components of cerebral blood velocity along with measures of arterial stiffness. Second, it presents a novel method that can readily be used at the bedside to assess stiffness of arteries perfusing the brain. Indeed, this method using ECG and TCD signals to obtain PAT, has the potential to provide reliable measurements in a myriad of clinical and dynamic settings, unlike duplex ultrasound and magnetic resonance imaging‐based measurements. This method is well suited for assessing changes in stiffness. However, the absolute values of PAT can less reliably be used to compare between individuals, due to the high degree of variability in cerebral blood vessel anatomy and the inclusion of the cardiac pre‐ejection period (Jones et al., **2021**).

Some limitations should also be further discussed. First, this study used TCD measurements of cerebral blood velocity to inform on changes in CBF, a parallel that requires stable arterial diameter (Aaslid et al., **1989**). Generally, a transient decrease in PCO_2_, such as those we observed during hemodialysis, would be expected to cause vasoconstriction and an increase in MCAv. However, a prolonged vasoconstrictive state resulting in reduced CBF could have contributed to the observed reduction in MCAv during hemodialysis. Were this the case, as PCO_2_ returned to baseline levels after hemodialysis, MCAv would have been expected to rise. Given that MCAv remained significantly reduced after hemodialysis, and that PCO_2_ remained within a physiological range throughout hemodialysis, it most likely was not a significant contributor to the decline in MCAv observed in this sample (Coverdale et al., **2014**; Verbree et al., **2014**). Second, our pilot study has a small sample size composed of relatively heterogeneous participants. While the small sample size limited the depth of characterization of pulsatile blood velocity during hemodialysis, our repeated measurement study design and adapted statistical model provided sufficient power to answer our research questions. The validity of our findings is further supported by the uniform response to hemodialysis in all participants for nearly all hemodynamic parameter of interest. Third, our findings may not be applicable to the posterior circulation of the brain, for which some studies have shown differential behavior (Labrecque et al., **2021**; Sato et al., **2011**, **2012**; Skow et al., **2013**). Fourth, while we showed no effect of sex on changes in MCAv, these findings should be interpreted with caution, as this pilot study was not designed to provide strong insight into the impact of sex pulsatile components of blood velocity and regional arterial stiffness (Lefferts et al., **2020**).

## 6 CONCLUSION

This study is the first, to the best of our knowledge, to consider the influence of both central and peripheral measures of BP on cerebral blood velocity and to provide a comprehensive account of pulsatile components of blood velocity during hemodialysis, along with measurements of regional arterial stiffness. Our findings support previous accounts of reduced ability to regulate cerebral blood velocity in CKD. Although this study identified reduced pulsatility of MCAv and cerebral artery stiffness during hemodialysis, which might be protective to the brain, there is a clear reduction of MCAv during hemodialysis, that can, in turn, contribute to the accelerated cognitive decline of ESKD patients on hemodialysis.

## AUTHOR CONTRIBUTIONS

Patrice Brassard and Mohsen Agharazii contributed to the original idea of the study; Mathilde Paré, Lawrence Labrecque, and Audrey Drapeau contributed to data collection; Hasan Obeid, Mathilde Paré, and Lawrence Labrecque contributed to data analyses; Mathilde Paré, Mohsen Agharazii, Patrice Brassard, and Hasan Obeid contributed to data interpretation; Mathilde Paré, Mohsen Agahrazii, and Patrice Brassard drafted the article. All persons designed as authors qualify as such and all those qualifying as authors are listed. All authors provided approval of the final article and agree to be accountable for all aspects of the work in ensuring that questions related to the accuracy or integrity of any part of the work are appropriately investigated and resolved.

## FUNDING INFORMATION

This study was funded with the support of the Agmen‐Université Laval Nephrology Research Chair. M.P. received a research internship grant from the Université Laval Faculty of Medicine. H.O. received a postdoctoral training scholarship from the Société québécoise d'hypertension artérielle (SQHA). L.L. received a doctoral training scholarship from the Société québécoise d'hypertension artérielle (SQHA).

## CONFLICT OF INTEREST STATEMENT

None.

## References

[phy215595-bib-0001] Aaslid, R. , Lindegaard, K. F. , Sorteberg, W. , & Nornes, H. (1989). Cerebral autoregulation dynamics in humans. Stroke, 20(1), 45–52. 10.1161/01.str.20.1.45 2492126

[phy215595-bib-0002] Ali, H. , Soliman, K. , Mohamed, M. M. , Daoud, A. , Shafiq, T. , Fülöp, T. , & Baharani, J. (2021). The effects of dialysis modality choice on cognitive functions in patients with end‐stage renal failure: A systematic review and meta‐analysis. International Urology and Nephrology, 53(1), 155–163. 10.1007/s11255-020-02603-x 32785817

[phy215595-bib-0003] Alwatban, M. R. , Aaron, S. E. , Kaufman, C. S. , Barnes, J. N. , Brassard, P. , Ward, J. L. , Miller, K. B. , Howery, A. J. , Labrecque, L. , & Billinger, S. A. (2021). Effects of age and sex on middle cerebral artery blood velocity and flow Pulsatility index across the adult lifespan. Journal of Applied Physiology, 130(6), 1675–1683. 10.1152/japplphysiol.00926.2020 33703940 PMC8285608

[phy215595-bib-0004] Avolio, A. , Kim, M. O. , Adji, A. , Gangoda, S. , Avadhanam, B. , Tan, I. , & Butlin, M. (2018). Cerebral Haemodynamics: Effects of systemic arterial pulsatile function and hypertension. Current Hypertension Reports, 20(3), 20. 10.1007/s11906-018-0822-x 29556793

[phy215595-bib-0005] Balestrini, C. S. , Al‐Khazraji, B. K. , Suskin, N. , & Shoemaker, J. K. (2020). Does vascular stiffness predict white matter Hyperintensity burden in ischemic heart disease with preserved ejection fraction? American Journal of Physiology. Heart and Circulatory Physiology, 318(6), H1401–H1409. 10.1152/ajpheart.00057.2020 32357114

[phy215595-bib-0006] Beutel, F. , Van Hoof, C. , Rottenberg, X. , Reesink, K. , & Hermeling, E. (2021). Pulse arrival time segmentation into cardiac and vascular intervals—Implications for pulse wave velocity and blood pressure estimation. IEEE Transactions on Biomedical Engineering, 68(9), 2810–2820. 33513094 10.1109/TBME.2021.3055154

[phy215595-bib-0007] Boero, R. , Pignataro, A. , Ferro, M. , & Quarello, F. (2001). Sympathetic nervous system and chronic renal failure. Clinical and Experimental Hypertension, 23(1–2), 69–75. 10.1081/ceh-100001198 11270590

[phy215595-bib-0008] Bowry, S. K. , Kircelli, F. , Himmele, R. , & Nigwekar, S. U. (2021). Blood‐incompatibility in haemodialysis: Alleviating inflammation and effects of coagulation. Clinical Kidney Journal, 14(Suppl 4), i59–i71. 10.1093/ckj/sfab185 34987786 PMC8711760

[phy215595-bib-0009] Brassard, P. , Labrecque, L. , Smirl, J. D. , Tymko, M. M. , Caldwell, H. G. , Hoiland, R. L. , Lucas, S. J. E. , Denault, A. Y. , Couture, E. J. , & Ainslie, P. N. (2021). Losing the dogmatic view of cerebral autoregulation. Physiological Reports, 9(15), e14982. 10.14814/phy2.14982 34323023 PMC8319534

[phy215595-bib-0010] Caldwell, H. G. , Carr, J. , Minhas, J. S. , Swenson, E. R. , & Ainslie, P. N. (2021). Acid‐base balance and cerebrovascular regulation. The Journal of Physiology, 599(24), 5337–5359. 10.1113/jp281517 34705265

[phy215595-bib-0011] Castro, P. , Azevedo, E. , & Sorond, F. (2018). Cerebral autoregulation in stroke. Current Atherosclerosis Reports, 20(8), 37. 10.1007/s11883-018-0739-5 29785667

[phy215595-bib-0012] Chung, S. , Jeong, H. S. , Choi, D. E. , Song, H. J. , Lim, Y. G. , Ham, J. Y. , Na, K. R. , & Lee, K. W. (2016). The impact of hemodialysis and arteriovenous access flow on extracranial hemodynamic changes in end‐stage renal disease patients. Journal of Korean Medical Science, 31(8), 1239–1245. 10.3346/jkms.2016.31.8.1239 27478334 PMC4951553

[phy215595-bib-0013] Coverdale, N. S. , Gati, J. S. , Opalevych, O. , Perrotta, A. , & Shoemaker, J. K. (2014). Cerebral blood flow velocity underestimates cerebral blood flow during modest hypercapnia and Hypocapnia. Journal of Applied Physiology, 117(10), 1090–1096. 10.1152/japplphysiol.00285.2014 25012027

[phy215595-bib-0014] Ferguson, G. G. , Eliasziw, M. , Barr, H. W. , Clagett, G. P. , Barnes, R. W. , Wallace, M. C. , Taylor, D. W. , Haynes, R. B. , Finan, J. W. , Hachinski, V. C. , & Barnett, H. J. (1999). The north American symptomatic carotid endarterectomy trial: Surgical results in 1415 patients. Stroke, 30(9), 1751–1758. 10.1161/01.str.30.9.1751 10471419

[phy215595-bib-0015] Fico, B. G. , Miller, K. B. , Rivera‐Rivera, L. A. , Corkery, A. T. , Pearson, A. G. , Eisenmann, N. A. , Howery, A. J. , Rowley, H. A. , Johnson, K. M. , Johnson, S. C. , Wieben, O. , & Barnes, J. N. (2022). The impact of aging on the association between aortic stiffness and cerebral Pulsatility index. Frontiers in Cardiovascular Medicine, 9, 821151. 10.3389/fcvm.2022.821151 35224051 PMC8863930

[phy215595-bib-0016] Findlay, M. D. , Dawson, J. , Dickie, D. A. , Forbes, K. P. , McGlynn, D. , Quinn, T. , & Mark, P. B. (2019). Investigating the relationship between cerebral blood flow and cognitive function in hemodialysis patients. Journal of the American Society of Nephrology, 30(1), 147–158. 10.1681/ASN.2018050462 30530658 PMC6317612

[phy215595-bib-0017] Fortier, C. , & Agharazii, M. (2016). Arterial stiffness gradient. Pulse (Basel), 3(3–4), 159–166. 10.1159/000438852 27195235 PMC4865077

[phy215595-bib-0018] Fu, X. , Huang, C. , Wong, K. S. , Chen, X. , & Gao, Q. (2016). A new method for cerebral arterial stiffness by measuring pulse wave velocity using transcranial Doppler. Journal of Atherosclerosis and Thrombosis, 23(8), 1004–1010. 10.5551/jat.33555 27052663 PMC7399300

[phy215595-bib-0019] Georgianos, P. I. , Sarafidis, P. A. , Malindretos, P. , Nikolaidis, P. , & Lasaridis, A. N. (2011). Hemodialysis reduces augmentation index but not aortic or brachial pulse wave velocity in dialysis‐requiring patients. American Journal of Nephrology, 34(5), 407–414. 10.1159/000331700 21934301

[phy215595-bib-0020] Ghoshal, S. , O'Connell, N. , Tegeler, C. , & Freedman, B. I. (2021). Cerebral hemodynamics in peritoneal dialysis versus intermittent hemodialysis: A transcranial Doppler pilot study. Peritoneal Dialysis International, 41(4), 417–422. 10.1177/0896860820953712 32909931

[phy215595-bib-0021] Giller, C. A. , & Aaslid, R. (1994). Estimates of pulse wave velocity and measurement of pulse transit time in the human cerebral circulation. Ultrasound in Medicine & Biology, 20(2), 101–105. 10.1016/0301-5629(94)90074-4 7912867

[phy215595-bib-0022] Go, A. S. , Chertow, G. M. , Fan, D. , McCulloch, C. E. , & Hsu, C. Y. (2004). Chronic kidney disease and the risks of death, cardiovascular events, and hospitalization. The New England Journal of Medicine, 351(13), 1296–1305. 10.1056/NEJMoa041031 15385656

[phy215595-bib-0023] Gottlieb, D. , Mildworf, B. , Rubinger, D. , & Melamed, E. (1987). The regional cerebral blood flow in patients under chronic hemodialytic treatment. Journal of Cerebral Blood Flow and Metabolism, 7(5), 659–661. 10.1038/jcbfm.1987.119 3654805

[phy215595-bib-0024] Hametner, B. , Wassertheurer, S. , Kropf, J. , Mayer, C. , Eber, B. , & Weber, T. (2013). Oscillometric estimation of aortic pulse wave velocity: Comparison with intra‐aortic catheter measurements. Blood Pressure Monitoring, 18(3), 173–176. 10.1097/MBP.0b013e3283614168 23571229

[phy215595-bib-0025] Hata, R. , Matsumoto, M. , Handa, N. , Terakawa, H. , Sugitani, Y. , & Kamada, T. (1994). Effects of hemodialysis on cerebral circulation evaluated by transcranial Doppler ultrasonography. Stroke, 25(2), 408–412. 10.1161/01.str.25.2.408 7905681

[phy215595-bib-0026] Hoiland, R. L. , Fisher, J. A. , & Ainslie, P. N. (2019). Regulation of the cerebral circulation by arterial carbon dioxide. Comprehensive Physiology, 9(3), 1101–1154. 10.1002/cphy.c180021 31187899

[phy215595-bib-0027] Holzer, H. , Marguc, K. , Pogglitsch, H. , Ott, E. , & Katschnig, H. (1981). The effects of Haemodialysis on cerebral blood flow. Proceedings of the European Dialysis and Transplant Association, 18, 126–132. 7329959

[phy215595-bib-0028] Jiang, X. L. , Wen, J. Q. , Zhang, L. J. , Zheng, G. , Li, X. , Zhang, Z. , Liu, Y. , Zheng, L. J. , Wu, L. , Chen, H. J. , Kong, X. , Luo, S. , Lu, G. M. , Ji, X. M. , & Zhang, Z. J. (2016). Cerebral blood flow changes in hemodialysis and peritoneal dialysis patients: An arterial‐spin labeling MR imaging. Metabolic Brain Disease, 31(4), 929–936. 10.1007/s11011-016-9829-7 27167984

[phy215595-bib-0029] Jones, J. D. , Castanho, P. , Bazira, P. , & Sanders, K. (2021). Anatomical variations of the circle of Willis and their prevalence, with a focus on the posterior communicating artery: A literature review and meta‐analysis. Clinical Anatomy, 34(7), 978–990. 10.1002/ca.23662 32713011

[phy215595-bib-0030] Labrecque, L. , Drapeau, A. , Rahimaly, K. , Imhoff, S. , & Brassard, P. (2021). Dynamic cerebral autoregulation and cerebrovascular carbon dioxide reactivity in middle and posterior cerebral arteries in young endurance‐trained women. Journal of Applied Physiology, 130(6), 1724–1735. 10.1152/japplphysiol.00963.2020 33955257

[phy215595-bib-0031] Lautt, W. W. (1989). Resistance or conductance for expression of arterial vascular tone. Microvascular Research, 37(2), 230–236. 10.1016/0026-2862(89)90040-x 2725343

[phy215595-bib-0032] Lefferts, W. K. , DeBlois, J. P. , Augustine, J. A. , Keller, A. P. , & Heffernan, K. S. (2020). Age, sex, and the vascular contributors to cerebral Pulsatility and pulsatile damping. Journal of Applied Physiology, 129(5), 1092–1101. 10.1152/japplphysiol.00500.2020 32940561 PMC7790130

[phy215595-bib-0033] London, G. M. (2018). Arterial stiffness in chronic kidney disease and end‐stage renal disease. Blood Purification, 45(1–3), 154–158. 10.1159/000485146, 10.1159/000485146 29478047

[phy215595-bib-0034] Lv, J. C. , & Zhang, L. X. (2019). Prevalence and disease burden of chronic kidney disease. Advances in Experimental Medicine and Biology, 1165, 3–15. 10.1007/978-981-13-8871-2_1 31399958

[phy215595-bib-0035] MacEwen, C. , Sutherland, S. , Daly, J. , Pugh, C. , & Tarassenko, L. (2017). Relationship between hypotension and cerebral ischemia during hemodialysis. Journal of the American Society of Nephrology, 28(8), 2511–2520. 10.1681/ASN.2016060704 28270412 PMC5533227

[phy215595-bib-0036] Mac‐Way, F. , Leboeuf, A. , & Agharazii, M. (2011). Arterial stiffness and dialysis calcium concentration. International Journal of Nephrology, 2011, 1–6. 10.4061/2011/839793 PMC3097079 21603117

[phy215595-bib-0037] Madero, M. , & Sarnak, M. J. (2011). Does hemodialysis hurt the brain? Seminars in Dialysis, 24(3), 266–268. 10.1111/j.1525-139X.2011.00857.x 21435001

[phy215595-bib-0038] Metry, G. , Spittle, M. , Rahmati, S. , Giller, C. , Giller, A. , Kaufman, A. , Schneditz, D. , Manno, E. , Brener, Z. , Boniece, I. , Ronco, F. , Ronco, C. , & Levin, N. W. (2002). Online monitoring of cerebral hemodynamics during hemodialysis. American Journal of Kidney Diseases, 40(5), 996–1004. 10.1053/ajkd.2002.36333 12407645

[phy215595-bib-0039] Mourad, A. , Carney, S. , Gillies, A. , Jones, B. , Nanra, R. , & Trevillian, P. (2004). Acute effect of haemodialysis on arterial stiffness: Membrane bioincompatibility? Nephrology, Dialysis, Transplantation, 19(11), 2797–2802. 10.1093/ndt/gfh443 15340092

[phy215595-bib-0040] Neumann, D. , Mau, W. , Wienke, A. , & Girndt, M. (2018). Peritoneal dialysis is associated with better cognitive function than hemodialysis over a one‐year course. Kidney International, 93(2), 430–438. 10.1016/j.kint.2017.07.022 29042081

[phy215595-bib-0041] Obeid, H. , Fortier, C. , Garneau, C. A. , Pare, M. , Boutouyrie, P. , Bruno, R. M. , Khettab, H. , Goupil, R. , & Agharazii, M. (2021). Radial‐digital pulse wave velocity: A noninvasive method for assessing stiffness of small conduit arteries. American Journal of Physiology. Heart and Circulatory Physiology, 320(4), H1361–h1369. 10.1152/ajpheart.00551.2020 33481697

[phy215595-bib-0064] Obeid, H. , Soulat, G. , Mousseaux, E. , Laurent, S. , Stergiopulos, N. , Boutouyrie, P. , & Segers, P. Numerical assessment and comparison of pulse wave velocity methods aiming at measuring aortic stiffness. Physiol Meas, 38(11), 1953–1967. 10.1088/1361-6579/aa905a 28968226

[phy215595-bib-0042] O'Rourke, M. F. , & Mancia, G. (1999). Arterial stiffness. Journal of Hypertension, 17(1), 1–4. 10.1097/00004872-199917010-00001 10100086

[phy215595-bib-0043] O'Rourke, M. F. , & Safar, M. E. (2005). Relationship between aortic stiffening and microvascular disease in brain and kidney. Hypertension, 46(1), 200–204. 10.1161/01.hyp.0000168052.00426.65 15911742

[phy215595-bib-0044] Parfrey, P. S. , & Foley, R. N. (1999). The clinical epidemiology of cardiac disease in chronic renal failure. Journal of the American Society of Nephrology, 10(7), 1606–1615. 10.1681/asn.V1071606 10405218

[phy215595-bib-0045] Polinder‐Bos, H. A. , Garcia, D. V. , Kuipers, J. , Elting, J. W. J. , Aries, M. J. H. , Krijnen, W. P. , Groen, H. , Willemsen, A. T. M. , van Laar, P. J. , Strijkert, F. , Luurtsema, G. , Slart, R. , Westerhuis, R. , Gansevoort, R. T. , Gaillard, C. , & Franssen, C. F. M. (2018). Hemodialysis induces an acute decline in cerebral blood flow in elderly patients. Journal of the American Society of Nephrology, 29(4), 1317–1325. 10.1681/ASN.2017101088 29496888 PMC5875962

[phy215595-bib-0046] Postiglione, A. , Faccenda, F. , Gallotta, G. , Rubba, P. , & Federico, S. (1991). Changes in middle cerebral artery blood velocity in uremic patients after hemodialysis. Stroke, 22(12), 1508–1511. 10.1161/01.str.22.12.1508 1962325

[phy215595-bib-0047] Rensma, S. P. , Stehouwer, C. D. A. , Van Boxtel, M. P. J. , Houben, A. J. H. M. , Berendschot, T. T. J. M. , Jansen, J. F. A. , Schalkwijk, C. G. , Verhey, F. R. J. , Kroon, A. A. , Henry, R. M. A. , Backes, W. H. , Dagnelie, P. C. , Van Dongen, M. C. J. M. , Eussen, S. J. P. M. , Bosma, H. , Köhler, S. , Reesink, K. D. , Schram, M. T. , & Van Sloten, T. T. (2020). Associations of arterial stiffness with cognitive performance, and the role of microvascular dysfunction. Hypertension, 75(6), 1607–1614. 10.1161/hypertensionaha.119.14307 32275192

[phy215595-bib-0048] Şahin Yildiz, B. , Şahin, A. , Başkurt Aladağ, N. , Arslan, G. , Kaptanoğullari, H. , Akın, İ. , & Yildiz, M. (2015). Acute effects of ultrafiltration on aortic mechanical properties determined by measurement of pulse wave velocity and pulse propagation time in hemodialysis patients. Anatolian Journal of Cardiology, 15(4), 313–317. 10.5152/akd.2014.5373 25413228 PMC5336841

[phy215595-bib-0049] Sato, K. , Fisher, J. P. , Seifert, T. , Overgaard, M. , Secher, N. H. , & Ogoh, S. (2012). Blood flow in internal carotid and vertebral arteries during orthostatic stress. Experimental Physiology, 97(12), 1272–1280. 10.1113/expphysiol.2012.064774 22689443

[phy215595-bib-0050] Sato, K. , Ogoh, S. , Hirasawa, A. , Oue, A. , & Sadamoto, T. (2011). The distribution of blood flow in the carotid and vertebral arteries during dynamic exercise in humans. The Journal of Physiology, 589(Pt 11), 2847–2856. 10.1113/jphysiol.2010.204461 21486813 PMC3112559

[phy215595-bib-0051] Shen, Z. , Ruan, Q. , Yu, Z. , & Sun, Z. (2017). Chronic kidney disease‐related physical frailty and cognitive impairment: A systemic review. Geriatrics & Gerontology International, 17(4), 529–544. 10.1111/ggi.12758 27240548

[phy215595-bib-0052] Skow, R. J. , MacKay, C. M. , Tymko, M. M. , Willie, C. K. , Smith, K. J. , Ainslie, P. N. , & Day, T. A. (2013). Differential cerebrovascular CO₂ reactivity in anterior and posterior cerebral circulations. Respiratory Physiology & Neurobiology, 189(1), 76–86. 10.1016/j.resp.2013.05.036 23774143

[phy215595-bib-0053] Slessarev, M. , Mahmoud, O. , Albakr, R. , Dorie, J. , Tamasi, T. , & McIntyre, C. W. (2021). Hemodialysis patients have impaired cerebrovascular reactivity to CO(_2_) compared to chronic kidney disease patients and healthy controls: A pilot study. Kidney International Reports, 6(7), 1868–1877. 10.1016/j.ekir.2021.04.005 34307981 PMC8258459

[phy215595-bib-0054] Sprick, J. D. , Nocera, J. R. , Hajjar, I. , O'Neill, W. C. , Bailey, J. , & Park, J. (2020). Cerebral blood flow regulation in end‐stage kidney disease. American Journal of Physiology. Renal Physiology, 319(5), F782–f791. 10.1152/ajprenal.00438.2020 32985235 PMC7789989

[phy215595-bib-0055] Stefanidis, I. , Bach, R. , Mertens, P. R. , Liakopoulos, V. , Liapi, G. , Mann, H. , & Heintz, B. (2005). Influence of hemodialysis on the mean blood flow velocity in the middle cerebral artery. Clinical Nephrology, 64(2), 129–137. 10.5414/cnp64129 16114789

[phy215595-bib-0056] Tian, X. , Guo, X. , Xia, X. , Yu, H. , Li, X. , & Jiang, A. (2019). The comparison of cognitive function and risk of dementia in CKD patients under peritoneal dialysis and hemodialysis: A PRISMA‐compliant systematic review and meta‐analysis. Medicine (Baltimore), 98(6), e14390. 10.1097/md.0000000000014390 30732180 PMC6380759

[phy215595-bib-0057] Van Sloten, T. T. , Protogerou, A. D. , Henry, R. M. A. , Schram, M. T. , Launer, L. J. , & Stehouwer, C. D. A. (2015). Association between arterial stiffness, cerebral small vessel disease and cognitive impairment: A systematic review and meta‐analysis. Neuroscience & Biobehavioral Reviews, 53, 121–130. 10.1016/j.neubiorev.2015.03.011 25827412 PMC5314721

[phy215595-bib-0058] Verbree, J. , Bronzwaer, A. S. , Ghariq, E. , Versluis, M. J. , Daemen, M. J. , van Buchem, M. A. , Dahan, A. , van Lieshout, J. J. , & van Osch, M. J. (2014). Assessment of middle cerebral artery diameter during Hypocapnia and hypercapnia in humans using ultra‐high‐field MRI. Journal of Applied Physiology, 117(10), 1084–1089. 10.1152/japplphysiol.00651.2014 25190741

[phy215595-bib-0063] Willie, C. K. , Colino, F. L. , Bailey, D. M. , Tzeng, Y. C. , Binsted, G. , Jones, L. W. , Haykowsky, M. J. , Bellapart, J. , Ogoh, S. , Smith, K. J. , Smirl, J. D. , Day, T. A. , Lucas, S. J. , Eller, L. K. , Ainslie, P. N. (2011). Utility of transcranial Doppler ultrasound for the integrative assessment of cerebrovascular function. J Neurosci Methods, 196, 221–237. 10.1016/j.jneumeth.2011.01.011 21276818

[phy215595-bib-0059] Willie, C. K. , Tzeng, Y. C. , Fisher, J. A. , & Ainslie, P. N. (2014). Integrative regulation of human brain blood flow. The Journal of Physiology, 592(5), 841–859. 10.1113/jphysiol.2013.268953 24396059 PMC3948549

[phy215595-bib-0060] Wolfgram, D. F. (2019). Intradialytic cerebral Hypoperfusion as mechanism for cognitive impairment in patients on hemodialysis. Journal of the American Society of Nephrology, 30(11), 2052–2058. 10.1681/asn.2019050461 31511363 PMC6830804

[phy215595-bib-0061] Wolfgram, D. F. , Szabo, A. , Murray, A. M. , & Whittle, J. (2015). Risk of dementia in peritoneal dialysis patients compared with hemodialysis patients. Peritoneal Dialysis International, 35(2), 189–198. 10.3747/pdi.2014.00213 25742686 PMC4406314

[phy215595-bib-0062] Zammit, A. R. , Katz, M. J. , Bitzer, M. , & Lipton, R. B. (2016). Cognitive impairment and dementia in older adults with chronic kidney disease. Alzheimer Disease & Associated Disorders, 30(4), 357–366. 10.1097/wad.0000000000000178 27861179 PMC5123843

